# HIST1H1C Regulates Interferon-β and Inhibits Influenza Virus Replication by Interacting with IRF3

**DOI:** 10.3389/fimmu.2017.00350

**Published:** 2017-03-24

**Authors:** Xiaokun Liu, Cha Yang, Yong Hu, Erming Lei, Xian Lin, Lianzhong Zhao, Zhong Zou, Anding Zhang, Hongbo Zhou, Huanchun Chen, Ping Qian, Meilin Jin

**Affiliations:** ^1^State Key Laboratory of Agricultural Microbiology, Huazhong Agricultural University, Wuhan, China; ^2^State Key Laboratory of Agricultural Microbiology, Key Laboratory of Agro-Microbiology Resources Development, College of Veterinary Medicine, Huazhong Agricultural University, Wuhan, China; ^3^Hubei Collaborative Innovation Center for Industrial Fermentation, Hubei University of Technology, Wuhan, China

**Keywords:** influenza A virus, HIST1H1C, NS2, IRF3, IFN-β

## Abstract

Influenza virus NS2 is well known for its role in viral ribonucleoprotein nuclear export; however, its function has not been fully understood. A recent study showed that NS2 might interact with HIST1H1C (H1C, H1.2). Histones have been found to affect influenza virus replication, such as the H2A, H2B, H3, and H4, but H1 has not been detected. Here, we found that H1C interacts with NS2 *via* its C-terminal in the nucleus and that H1C affects influenza virus replication. The H1N1 influenza virus replicates better in H1C knockout A549 cells compared to wild-type A549 cells, primarily because of the regulation of H1C on interferon-β (IFN-β). Further studies showed that the H1C phosphorylation mutant (T146A) decreases IFN-β, while H1C methylation mutants (K34A, K187A) increases IFN-β by releasing the nucleosome and promoting IRF3 binding to the IFN-β promoter. Interestingly, NS2 interacts with H1C, which reduces H1C–IRF3 interaction and results in the inhibition of IFN-β enhanced by H1C. In summary, our study reveals a novel function of H1C to regulate IFN-β and uncovers an underlying mechanism, which suggests H1C plays a role in epigenetic regulation. Moreover, our results suggest a novel mechanism for the influenza virus to antagonize the innate immune response by NS2.

## Introduction

The influenza virus NS2, whose most important role is to regulate viral ribonucleoprotein nuclear export, affects influenza virus polymerase activity. However, its function has not been fully understood, and in-depth studies are needed to further elucidate its functions and the mechanism. A recent study on the influenza A virus NS2 demonstrated that it potentially interacts with host factors, including HIS1H1C (H1C, H1.2) (1). Histones have been recognized for their abundant effects on the host cell and pathogens. Hoeksema et al. found that histones H3 and H4 neutralize influenza virus in a more robust manner compared to H2A and H2B (2), while histone H1 has not been detected. These findings indicate that H1C may affect influenza virus replication.

H1C belongs to the histone H1 family, interacts with linker DNA between nucleosomes, and plays a role in the compaction of chromatin into higher order structures (3, 4). Previous studies have demonstrated that H1C can be phosphorylated by DNA-dependent protein kinase at the T146 amino residue (5) and methylated by euchromatic histone-lysine *N*-methyltransferase (EHMT1/2) at the K34 and K187 amino residues (6). EHMT1/2 are components of the E2F6 complex, which repress gene transcription and participate in regulation of the cell cycle (7, 8). H1C participates in apoptosis, which is mainly induced by DNA damage (9–11). Recently, H1C phosphorylation was found to regulate p53 acetylation and plays a role in DNA damage repair (12); however, the function of H1C methylation is still unknown.

Typically, viruses can produce pathogen-associated molecular patterns (PAMPs) during replication (13, 14); PAMPs then interact with various pattern recognition receptors (PRRs) in the cell membrane and cytoplasm. Following PAMP recognition, PRR-induced signal transduction activates antiviral genes transcriptions (13, 15, 16). Influenza virus can be recognized by RIG-I and activates the innate immune response, ultimately inducing interferon-β (IFN-β) production (17). However, influenza virus can also inhibit IFN-β *via* different mechanisms; for example, NS1 protein can target tripartite motif-containing protein 25 (TRIM25) and riplets ubiquitin E3 ligases in a species-specific manner to drive the inhibition of RIG-I ubiquitination and antiviral IFN production (18). Recently, histones have been found to participate in the regulation of innate immunity; for instance, extrachromosomal histone H2B interacts with IFN-β promoter stimulator 1, which is engaged in the signaling pathway initiated by dsDNA to trigger antiviral innate immune responses (19). However, the effect of H1C on innate immune response has not been revealed.

Here, we performed experiments to investigate the effect of H1C on influenza virus replication and found that H1C inhibits H1N1 influenza virus replication. The virus replicates better in H1C knockout (H1C-KO) A549 cells compared to wild-type cells. Further data showed that H1C is involved in the innate immune response and regulates IFN-β by interacting with IRF3. Interestingly, H1C interacts with NS2 *via* its C-domain in the nucleus, and NS2 reduces H1C–IRF3 interaction and inhibits IFN-β induced by H1C.

## Materials and Methods

### Cells and Viruses

Human embryonic kidney 293 T (HEK293T) cells, Henrietta Lacks strain of cancer cells (HeLa), and adenocarcinomic human alveolar basal epithelial cells (A549) are maintained in DMEM (Gibco, New York, NY, USA), 1640 and F12 (HyClone, Beijing, China) medium supplemented with 10% fetal bovine serum, and cultured at 37°C under 5% CO_2_. Influenza A virus H1N1 [A/WSN/1933(H1N1)] (WSN) was obtained by reverse genetics as described by Hoffmann and Webster (20) and maintained by our laboratory. Sendai virus (Sev) was kindly provided by Professor Zhengfan Jiang (Institute of Life & Science, Peking University, China).

### Plasmids and Small Interfering RNA (siRNA)

The H1C open reading frame (NCBI, NM_005319.3) was amplified by PCR using the primers H1C-*Eco*RI (AAAgaattcaATGTCCGAGACTGCTCCTGCCG) and H1C-*Bam*HI (AAAggatccTTTCTTCTTGGGCGCCGCCTTCT) and cloned into the p3Xflag-CMV vector. The H1C mutants (K34A, T146A, K187A, H1C-NT, and H1C-CT) were constructed by PCR using primer star (Takara, Tokyo). The GFP fusion plasmids GFP-H1C, GFP-H1C-NT, and GFP-H1C-CT were constructed as described above. The nucleotide sequence of the NS2 protein of A/WSN/1933(H1N1) was synthesized according to a sequence in GenBank (U13682.1) and cloned into the pCAGGS-HA vector. The plasmids pDsRed-NS2, pDsRed-NS2-NT, and pDsRed-NS2-CT were constructed as described above. siRNA targeted to H1C (si-H1C) was used and the sequence was as follows: 5′-UUUUUCUCCACAUCAUAGCCG-3′; a non-target siRNA was used as negative control (si-NC), and the sequence was as follows: 5′-UUCUCCGAACGUGUCACGUTT-3′; a siRNA targeted to GAPDH was used as a positive control (si-GAPDH), and the sequence was as follows: 5′-UGACCUCAACUACAUGGUUTT-3′.

### Transfection

Transfection was performed using lip2000 reagent according to the manufacturer’s instructions. Both plasmids or siRNA and lip2000 reagent were diluted with equal value of opti-MEM medium and kept for 5 min at room temperature, following by mixing them generally, Next, the mixture was added into plates after 20 min, and cells were cultured with fresh medium with or without serum at 6 h post-transfection.

### Immunofluorescence (IF) Confocal Microscopy

Immunofluorescence confocal microscopy was performed as described (21). HeLa or A549 cells were fixed with 4% paraformaldehyde for 10 min, treated with 2‰ Triton-X100 for 10 min, and incubated with 1% BSA for 1 h at room temperature. The sample was then incubated with specific antibodies [anti-Flag mouse monoclonal antibody (Sigma, F3165, USA), anti-HA rabbit polyclonal antibody (PMK Bio., PMK013C, China), anti-NS2 mouse polyclonal antibody (prepared by our lab), and anti-H1C rabbit polyclonal antibody (Proteintech, 19649-1-AP, China)] for 2 h followed by incubation with goat anti-mouse FITC-labeled and goat anti-rabbit Cy3-labeled secondary antibodies (KPL, No. 172-1806-1, No. 072-01-15-06-1, USA). Finally, images were acquired using confocal microscopy (LSM510 ZEISS, Germany).

### Co-Immunoprecipitation (Co-IP)

A549 cells were infected with influenza virus at an MOI of 10 and lysed at 10 h post-infection. Co-IP was performed as described (22) using the NS2 polyclonal antibody, and sodium dodecyl sulfate-polyacrylamide gel electrophoresis (SDS-PAGE) and western blotting analyses were performed. To demonstrate the H1C–NS2 interaction, HEK293T cells were co-transfected with Flag-H1C and HA-NS2, and Co-IP was performed as described above using an anti-HA antibody and analyzed using western blotting using anti-Flag antibody. To detect the H1C–IRF3 interaction, HEK293T cells were transfected with HA-IRF3 and Flag-H1C or its mutants and infected with Sev at 24 h post-transfection and lysed for IP at 12 h post-infection using an anti-Flag monoclonal antibody.

### Subcellular Fractions Extraction

Subcellular fractions were extracted as described (23). A total of 10^6^ HEK293T or A549 cells treated accordingly were harvested and lysed with 100 µL of cytoplasmic extraction buffer on ice for 20 min followed by the addition of NP-40 (Amresco, Solon, USA) at a final concentration of 0.5%, and the sample was vortexed for 15 s. Next, the sample was centrifuged for 10 min at 3,500 *g* at 4°C, and the supernatant was stored as the cytoplasm fraction. The pellet was dissolved in 80 µL nucleus extraction buffer and incubated on ice for 10 min followed by centrifugation for 10 min at 14,000 *g* at 4°C. Finally, the supernatant was collected and stored as the nuclei fraction until further analyses.

### RNA Quantitation RT-PCR Analysis

Cells were lysed with TRIzol Reagent (Invitrogen, USA), and the total RNA was extracted according to the manufacturer’s instructions. Two micrograms of RNA was used to generate cDNA using reverse transcriptase (AMV XL TaKaRa, Tokyo) with oligo-dT-18T. Then, the cDNA was used as a template for real-time PCR (ABI Vii7A, USA), and the level of the target gene was normalized to the housekeeping gene glyceraldehyde 3-phosphate dehydrogenase (GAPDH) or β-actin. The sequences of primers used for RT-PCR are given in Table 1.

**Table 1:** **Primers used for RT-PCR**.

Gene name	Forward sequences (5′–3′)	Reverse sequences (5′–3′)
H1C	GGCAGCCTCCGGGGAAGC	CGGCTTCTTCGCTTTCTTCGGTG
NP	GCGTTCAGCCCACTTTCTCG	GGGTTCGTTGCCTTTTCGTC
GAPDH	GCTAAGGCTGTGGGCAAGG	GGAGGAGTGGGTGTCGCTG
β-actin	CAGGGCGTGATGGTGGGCA	CAAACATCATCTGGGTCATCTTCTC
Interferon-β	GCTCCTGTGGCAATTGAATGG	TTGGCCTTCAGGTAATGCAG
IL-6	ACAGCCACTCACCTCTTCAGAAC	GCTCTGGCTTGTTCCTCACTACTC
IL-8	TGCAGCTCTGTGTGAAGGTG	CAGCCCTCTTCAAAAACTTCTCC
TNF-α	CAGGCGGTGCTTGTTCC	AAGAGGACCTGGGAGTAGATGA
OASL	GCCTTCTCTTCCCAACTCCC	AGGCATAGATGGTTAGAAGTTCAAGA
MX1	CCGAGGGAGACAGGACCAT	CGTGGCCTTTCCTTCCTCC
CXCL10	ATTTGCTGCCTTATCTTTCTGACTCTA	TGGCCTTCGATTCTGGATTCA

*The gene sequences are downloaded from NCBI, and primers are designed by Primer 5*.

### Viral Growth Curve Measurement

A549 cells in 12-well plates were transfected with plasmids (1 µg) or siRNA (40 pmol) and then infected with influenza virus at an MOI of 0.01. The samples were harvested at 12, 24, 36, and 48 h post-infection. A plaque assay was performed as described (24), and the cells were cultured until the plaques had distinctly formed. Finally, the number of plaques was quantified.

### Western Blotting Analysis

Equal amount protein of each sample was loaded for SDS-PAGE, and protein was transferred from the gel onto a nitrocellulose filter membrane, followed by blotting with specific antibodies. Finally, a Chemiluminescence Imaging System (DNR, USA) was used for the analysis and images were acquired.

### Chromatin Immunoprecipitation and Quantitation PCR (ChIP-qPCR)

Human embryonic kidney 293 T cells were co-transfected with HA-IRF3 and Flag-H1C or its mutants and ChIP-qPCR was performed as described (25) using an anti-HA monoclonal antibody. A mouse monoclonal poly II antibody (EPITOMICS, 2035-1, CA, USA) served as the positive control, and a blank mouse IgG (ZKCY 161025, Beijing China) served as the negative control. The primer sequences (IFN-β promoter) used in this experiment were as follows: forward, 5′-TAGGAAAACTGAAAGGGAGAAG-3′; reverse, 5′-TGTCGCCTACTACCTGTTGTG-3′.

### Construction of the H1C-KO Cell Line

The H1C-KO A549 cell line (A549-H1C-KO) was constructed using the CRISPR/Cas9 system (26), and the guide sequence was as follows: 5′- AACCAATGTCACCGGCGCCGGCC- 3′, 5′- TTGGTTACAGTGGCCGCGGCCGG- 3′. A549 cells were transfected with px335-H1C plasmid and cultured for 2 days. This was repeated twice, and the cells were digested, highly diluted, and cultured in a 48-well plate until the cells had grown as a monolayer. Finally, these cells were analyzed by PCR and western blotting to investigate whether H1C was knocked out, and the positive cells were used for further experiments.

### Statistical Analysis

The data were presented as the mean ± SD of three independent experiments, and statistical significance was determined using two-way ANOVA. A *p*-value less than 0.05 was considered statistically significant (*p < 0.05, **p < 0.01, ***p < 0.001).

## Results

### NS2 Interacts with H1C

**Figure 1:**
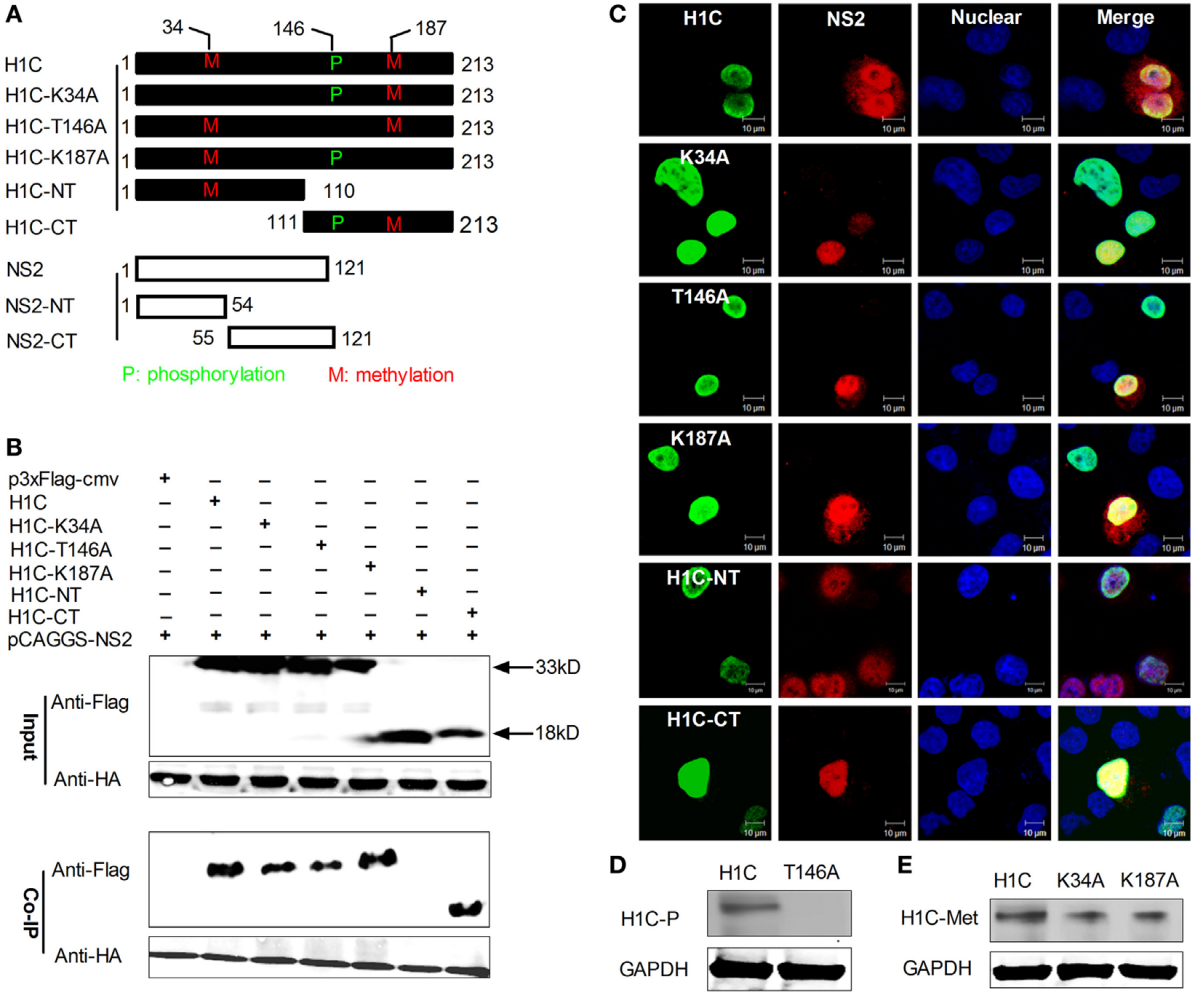
**Investigation of the interaction between H1C and NS2**. **(A)** A schematic diagram of the H1C and NS2 substitutions, the phosphorylation site (T146) or methylation site (K34 and K187) of H1C were mutated, and H1C was divided into the N-terminal and C-terminal regions. NS2 was also divided into the N-terminal and C-terminal regions. **(B)** Human embryonic kidney 293 T (HEK293T) cells were transfected with HA-NS2 and Flag-H1C or its mutants, and a co-immunoprecipitation (Co-IP) experiment was performed using anti-HA antibody to detect the interactions. **(C)** HeLa cells cultured on slides were co-transfected with HA-NS2 and Flag-H1C or its mutants, and immunofluorescence confocal microscopy was performed 24 h later using anti-Flag mouse monoclonal antibody and anti-HA rabbit polyclonal antibody followed by immunostaining with FITC-labeled goat anti-mouse secondary antibody and Cy3-labeled goat anti-rabbit antibody. **(D)** Detection of the phosphorylation levels of the H1C phosphorylation mutant. H1C or H1C-T146A expressed in HEK293T cells were immune-precipitated using the anti-flag monoclonal antibody (Sigma, F1804, USA) and analyzed by western blotting using the threonine phosphorylation antibody. **(E)** Detection of the methylation levels of H1C methylation mutants. H1C, H1C–K34A, or H1C–K187A expressed in HEK293T cells were immune-precipitated using the anti-flag antibody and detected by western blotting using the methylation antibody.

To confirm the interaction between NS2 and H1C, HA-NS2 and Flag-H1C were co-expressed in HEK293T cells, and a Co-IP experiment was performed using the anti-HA monoclonal antibody. The data showed that NS2 really interacted with H1C. To further investigate the interaction domains of them, H1C was divided into regions, the N-terminal (H1C-NT) and C-terminal (H1C-CT). In addition, the phosphorylation (T146A) or methylation (K34A, K187A) mutants were generated. Furthermore, NS2 was divided into N-terminal (NS2-NT) and C-terminal (NS2-CT) regions (Figure 1A). When using wild-type HA-NS2 to immune-precipitate Flag-H1C and its mutants, it could only interact with the C-terminal of H1C but had no interaction with the N-terminal of H1C, indicating that H1C interacted with NS2 *via* its C-domain. Moreover, when tested with H1C phosphorylation and methylation mutants, NS2 showed interactions with all these mutants; the T146A mutation decreased the interaction compared with H1C wild type, while the K187A mutation increased the interaction. The K34A mutants slightly weakened this interaction (Figure 1B). To investigate whether NS2 and H1C or its mutants co-localized in the cell, HA-NS2 and Flag-H1C or its mutants were co-expressed in HeLa cells and IF confocal microscopy was performed as described previously. The data showed that H1C and its mutants localized in the nucleus, and NS2 localized in the whole cell but predominantly in the nucleus. NS2 showed good co-localization with H1C and K34A, T146A, K187A, and H1C-CT mutants in the nucleus but little co-localization with H1C-NT, although the two proteins were both localized in the nucleus (Figure 1C). In addition, to confirm the phosphorylation or methylation mutation, the phosphorylation and methylation levels of the proteins were detected and indicated that the mutation of the T146 residue resulted in a loss of H1C phosphorylation (Figure 1D). However, the methylation ability was weakened when the K34 or K187 residues were individually mutated (Figure 1E).

**Figure 2:**
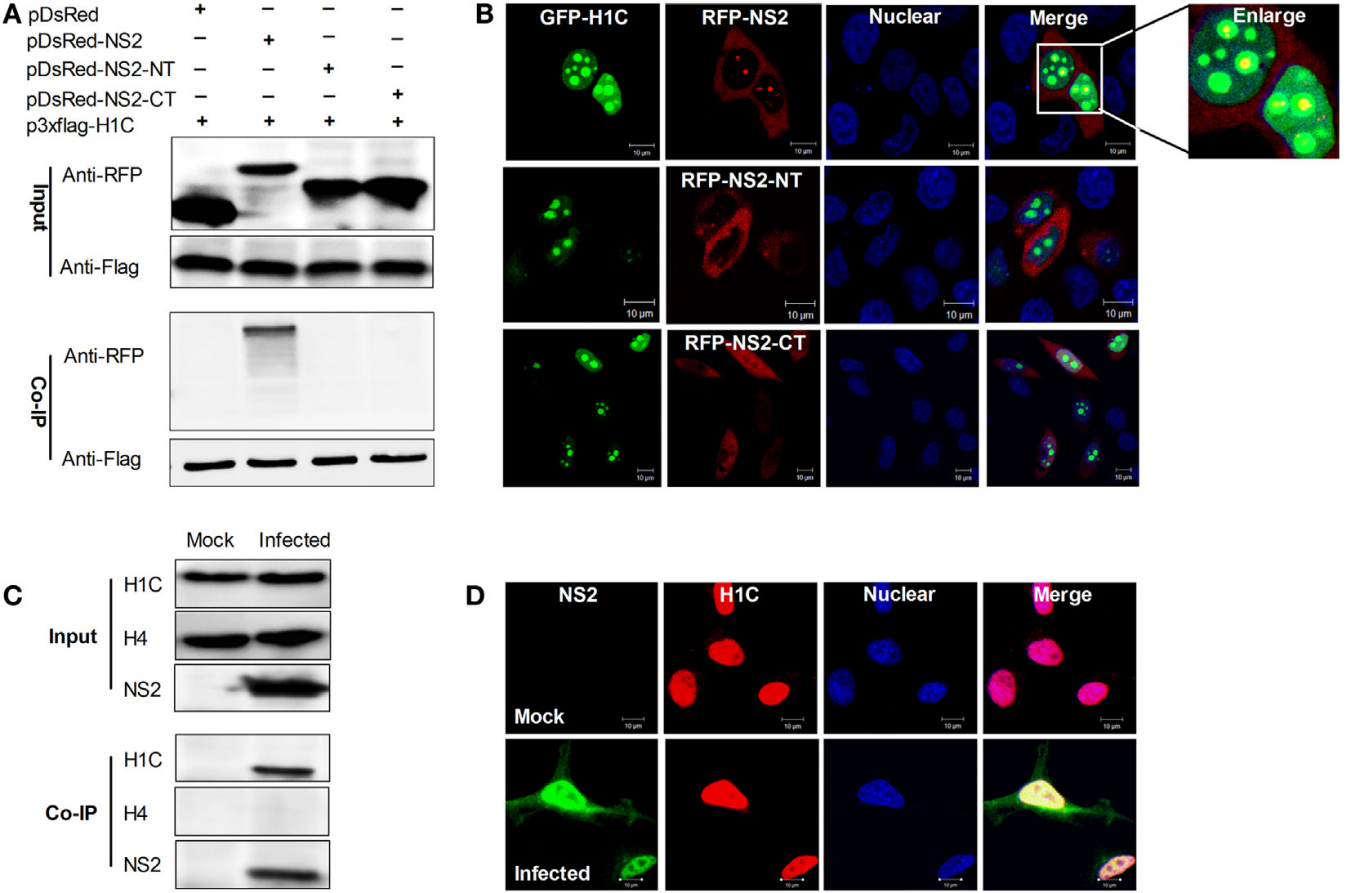
**Investigation of the interaction domain of NS2 and the interaction between NS2 and endogenous H1C**. **(A)** Human embryonic kidney 293 T cells were transfected with Flag-H1C and RFP-NS2 or its mutants, and a co-immunoprecipitation (Co-IP) was performed to detect interactions using the anti-flag antibody. An anti-RFP antibody was used to detect pDsRed-NS2 or its mutants. **(B)** HeLa cells cultured on slides were co-transfected with pEGFP-H1C and pDsRed-NS2, pDsRed-NS2-NT, or pDsRed-NS2-CT, and confocal microscopy was performed 24 h later to detect the co-localization. **(C)** A549 cells were infected with influenza virus for 10 h (WSN,MOI = 10), and an IP experiment was performed using an anti-NS2 antibody to detected the interaction between NS2 and endogenous H1C. Histone H4 (H4) served as a negative control. **(D)** A549 cells were treated as described above, and confocal microscopy was performed using the anti-NS2 mouse polyclonal antibody and anti-H1C rabbit polyclonal antibody followed by immunostaining with FITC-labeled goat anti-mouse secondary antibody and Cy3-labeled goat anti-rabbit antibody to detect its co-localization.

The above data showed that H1C interacted with NS2 *via* its C-domain, but by which domain does NS2 interacts with H1C? To answer this question, NS2 was divided into the NT and CT domains and fused with an RFP tag because the constructs were too small to be detected using western blotting. Next, Flag-H1C and RFP-NS2, RFP-NS2-NT or RFP-NS2-CT were co-expressed in HEK293T cells, and Co-IP experiments were performed, and these results showed that only full-length NS2 could interact with H1C (Figure 2A). Again, confocal microscopy was performed to observe the co-localization of the proteins, demonstrating that GFP tagged H1C was granularly distributed in the nucleus, and co-localized with RFP fused NS2. The NS2-NT lost the ability to localize in the nucleus compared with wild-type NS2, while NS2-CT was distributed throughout the cell, and both of the proteins showed little co-localization with H1C (Figure 2B). Moreover, to investigate the interaction between NS2 and endogenous H1C during infection, A549 cells were infected with influenza virus (WSN), and a Co-IP experiment was performed using the NS2 polyclonal antibody. The data revealed that NS2 interacted with endogenous H1C during infection. To further confirm whether this interaction is specific, another histone protein was used for detection and the data showed that NS2 had no interaction with histone H4, which indicated that NS2 specifically interacted with H1C (Figure 2C). In addition, results obtained from confocal microscopy showed that the proteins were co-localized in the nucleus during infection (Figure 2D).

### H1C Regulates Influenza Virus Replication

**Figure 3:**
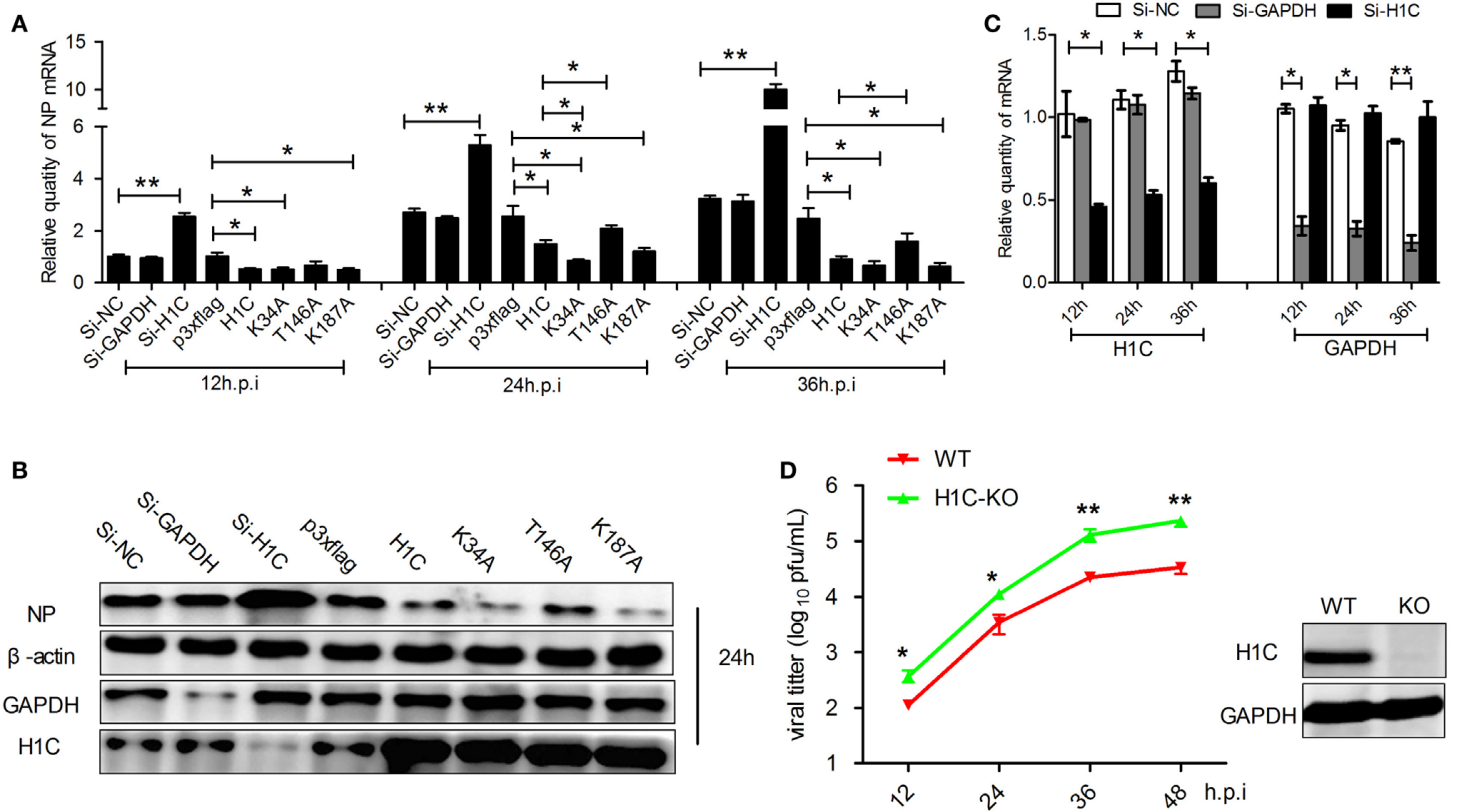
**The effect of H1C on influenza A viral replication and proliferation in A549 cells**. **(A)** A549 cells transfected with small interfering RNA (siRNA), H1C or its mutants were infected (WSN, MOI = 0.01); and the samples were collected at 12, 24, and 36 h, respectively. Next, real-time PCR was performed to detect the NP mRNA levels. A non-target siRNA (Si-NC) and the siRNA targeted to GAPDH (Si-GAPDH) were used as negative or positive controls, respectively, to detect the silencing specificity of H1C. **(B)** Cells were treated as described above, and western blotting analyses were performed to detect the NP expression level. **(C)** Detection of H1C silencing using real-time PCR. **(D)** One step viral growth curve detection on A549 or A549-H1C knockout (H1C-KO) cells. A549 and A549-H1C-KO cells were infected with influenza virus (WSN, MOI = 0.01). The supernatants were then collected at 12, 24, 36, and 48 h post-infection, and the viral titers were detected using the plaque assay on MDCK cells (*p < 0.05, **p < 0.01, the data were generated from three independent experiments).

**Figure 4:**
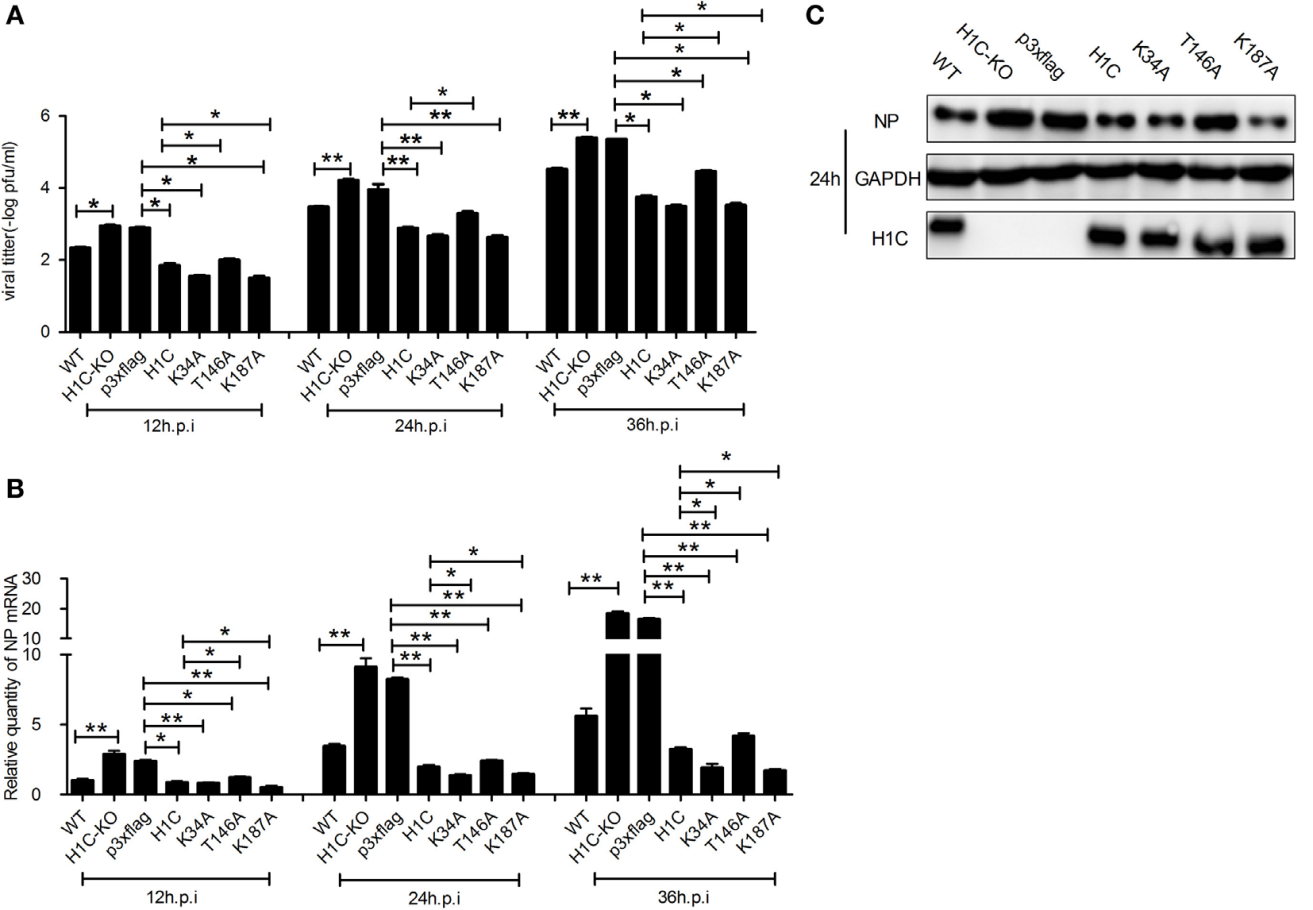
**Investigation of the effect of H1C on influenza A viral replication and proliferation in A549-H1C-KO cells**. **(A)** A549-H1C-KO cells transfected with H1C or its mutants were then infected with influenza virus (WSN, MOI = 0.01). The supernatants were collected at 12, 24, and 36 h post-infection, and the viral titers were detected using plaque assay on MDCK cells. **(B)** A549-H1C-KO cells were treated as described above, and the RNA was collected at 12, 24, and 36 h post-infection, and NP mRNA was detected using real-time PCR. **(C)** Cells were treated as described above, and western blotting analyses were conducted to detect the NP expression levels (*p < 0.05, **p < 0.01, the data were generated from three independent experiments).

As H1C interacts with NS2 and histones play a role in influenza virus replication, suggesting that H1C may involve in the regulation of influenza virus replication. To investigate this question, A549 cells were transfected with siRNA, H1C or its mutants and infected with influenza virus, and NP mRNA and expression levels were detected. The result showed that NP mRNA was significantly increased when silencing H1C but significantly decreased when overexpressing H1C. Interestingly, the K34A and K187A mutants reduced virus replication more significantly compared with H1C, while the T146A mutant reduced the inhibition of H1C on viral replication (Figure 3A). To further confirm these observations, western blotting analyses were performed, and the results showed that the NP expression level of the H1C-silenced group was much higher compared to the Si-NC group, while overexpression of H1C significantly reduced NP expression levels compared with the vector group. These findings were consistent with the results of the mRNA levels (Figure 3B). The silencing efficiency of H1C was detected using real-time PCR, and it showed that H1C was specifically silenced during infection (Figure 3C); the Si-NC and Si-GAPDH groups were served as negative or positive controls. To better understand the effect of H1C on influenza A virus, H1C-KO A549 cells (A549-H1C-KO) were generated using Crisp/cas9. In addition, the virus growth curve was measured in both A549 wild-type and knockout cell lines. Expectedly, virus proliferated more robustly in A549-H1C-KO cells compared to A549 wild-type cells (A549-WT) (Figure 3D). The H1C expression was detected by western blotting, and it showed that H1C was successfully knockout. However, when H1C was expressed in A549-H1C-KO cells, the virus titer was significantly inhibited. Moreover, K34A and K187A mutations significantly enhanced the inhibitory ability of H1C on virus titer, while the T146A mutation relieved this inhibition (Figure 4A). Furthermore, NP mRNA levels were also detected, and these results were consistent with that of the virus titer (Figure 4B). In addition, a similar result was found when examining the NP expression levels by western blotting (Figure 4C).

### H1C Is Involved in the Regulation of IFN-β

**Figure 5:**
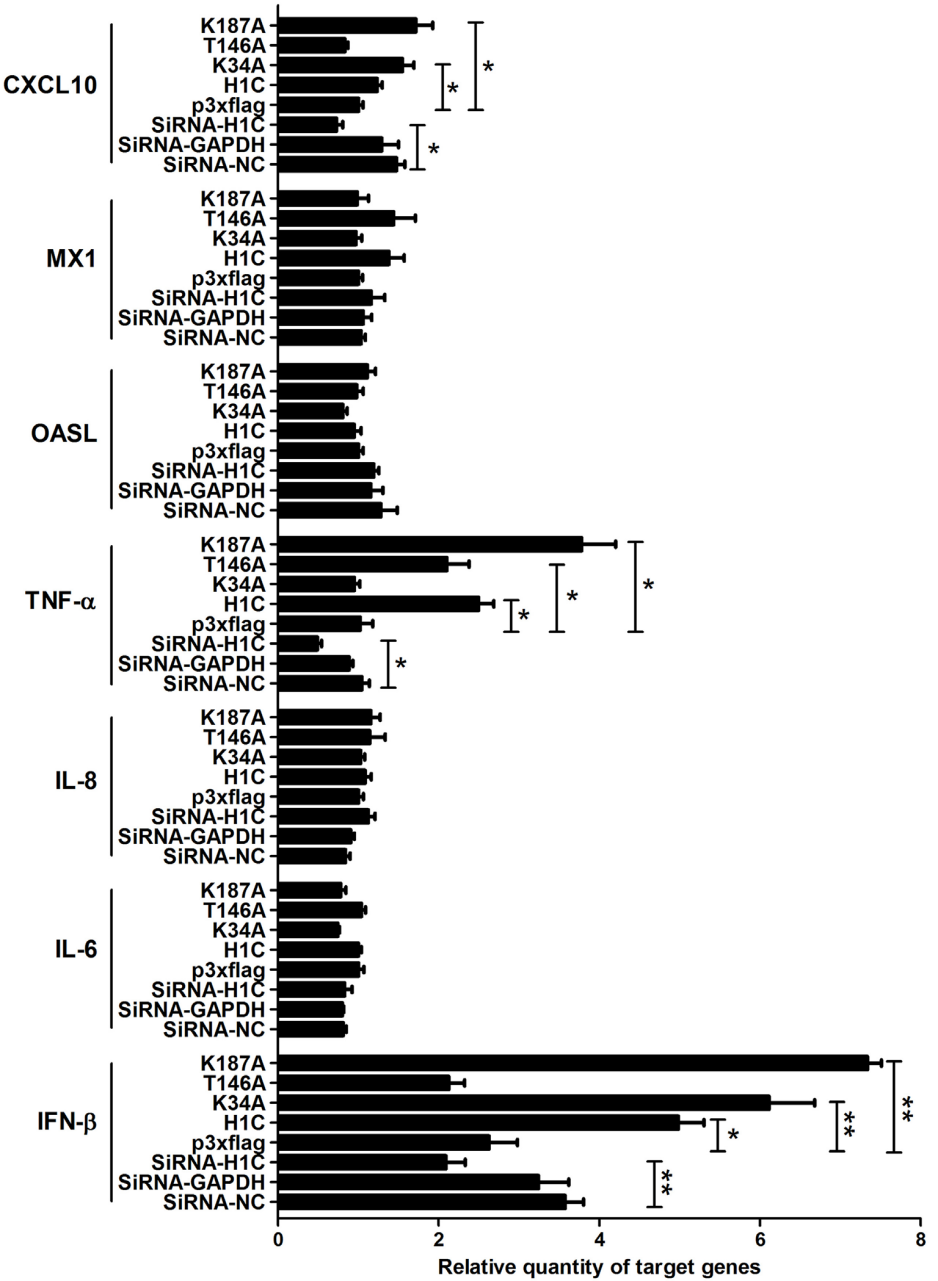
**Analysis of the effects of H1C on cytokines and chemokines**. A549 cells were transfected with small interfering RNA (siRNA), H1C or its mutants and were harvested at 24 h later. The mRNA of target genes [interferon-β (IFN-β), IL-6, IL-8, TNF-α, OASL, MX1, and CXCL10] were detected using real-time PCR, a non-target siRNA (Si-NC), and the siRNA targeted to GAPDH (Si-GAPDH) were used as a negative or positive control to detect the silencing specificity of H1C (*p < 0.05, **p < 0.01, the data were generated from three independent experiments).

**Figure 6:**
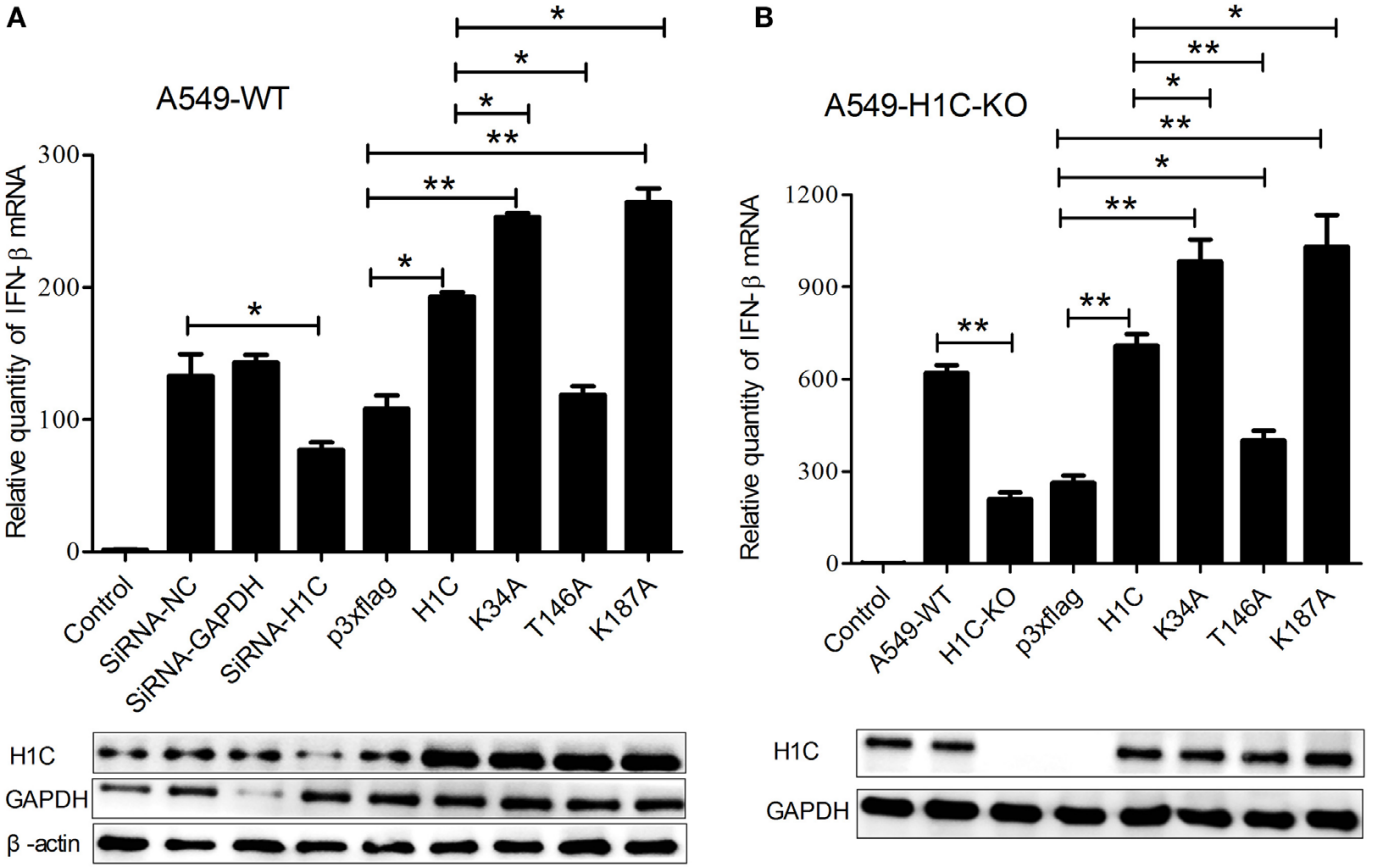
**Investigation of the effects of H1C on interferon-β (IFN-β) in A549 or A549-H1C-KO cells**. **(A)** A549 cells transfected with small interfering RNA (siRNA), H1C or its mutants were stimulated with poly(I: C) (20 nmol/mL) for 6 h, and IFN-β mRNA levels were detected using real-time PCR. A non-target siRNA (Si-NC) and the siRNA targeted to GAPDH (Si-GAPDH) were used as the negative or positive control to detect the silencing specificity of H1C. **(B)** A549-H1C-KO cells were overexpressed with H1C or its mutants and stimulated with poly (I:C) as described above, and IFN-β mRNA levels were then detected. H1C silencing or expression levels were detected by western blotting (*p < 0.05, **p < 0.01, the data were generated from three independent experiments).

To explore the mechanism underlying H1C inhibition of influenza virus replication, the virus-related cytokines and chemokines were detected because they play an important role in the antiviral process, and histones might be involved in the innate immune response. A549 cells were transfected with siRNA, H1C or its mutants, and RT-PCR was performed to detect those genes mRNA levels. These data showed that H1C significantly upregulated IFN-β and K34A, and K187A mutants enhanced this ability, while the T146A mutation significantly decreased this upregulation. However, silencing or overexpressing H1C or its mutants showed little effect on MX1. In addition, H1C had little effect on OASL, IL-8, and IL-6 production. Moreover, TNF-α was significantly increased when H1C, K34A, and K187A mutants were overexpressed and were significantly inhibited when H1C was silenced. In addition, overexpression of H1C increased CXCL10 expression (Figure 5). Because H1C had a more significant effect on IFN-β than other cytokines and IFN-β plays an important role in the antiviral process, further experiments were performed to determine how H1C regulates IFN-β. The IFN-β mRNA levels were detected when H1C was silenced or overexpressed on A549 cells, and the data showed that IFN-β production was reduced when H1C was silenced, while it was increased when H1C was overexpressed. Interestingly, K34A and K187A substitutions enhanced the ability of H1C to induce IFN-β, while the T146A mutation significantly inhibited the ability of H1C to induce IFN-β compared with H1C (Figure 6A), which was consistent with the results of the virus replication. To better understand the role of T146, K34, and K187 residues on the regulation of IFN-β, A549-H1C-KO cells were used. The data showed that IFN-β level in A549-H1C-KO cells was much lower than A549-WT cells, and it was significantly increased to a level that was similar to wild-type cells when H1C was overexpressed. Expectedly, K34A and K187A mutations significantly enhanced the ability of H1C to induce IFN-β, while T146A mutations significantly inhibited this induction (Figure 6B). Silencing or expression levels of H1C were detected using western blotting analyses.

**Figure 7:**
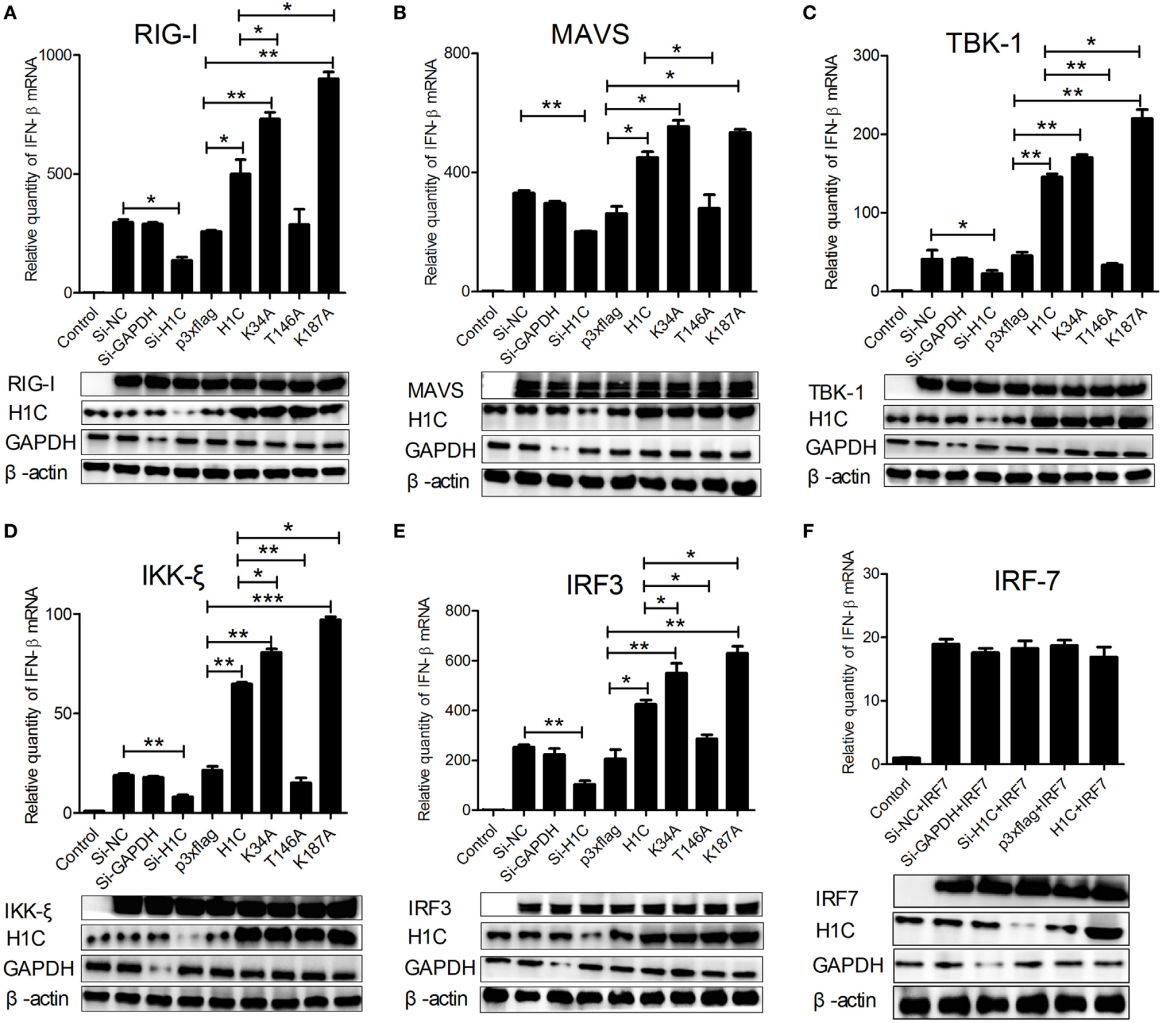
**Interferon-β (IFN-β)-inducing signaling pathway analysis**. **(A)** A549 cells cultured in 12-well plates were co-transfected with RIG-I (1 µg), H1C (1 µg) or its mutants, and small interfering RNA (siRNA) (40 pmol), and the IFN-β mRNA levels were detected by RT-PCR 24 h later. A non-target siRNA (Si-NC) and the siRNA targeted to GAPDH (Si-GAPDH) were used as a negative or positive control to detect the silencing specificity of H1C. **(B)** Analysis of the effect of H1C or its mutants on IFN-β stimulated by MAVS using real-time PCR. **(C)** Analysis of the effect of H1C or its mutants on IFN-β stimulated by TBK-1 using real-time PCR. **(D)** Analysis of the effect of H1C or its mutants on IFN-β stimulated by IKK-ξ using real-time PCR. **(E)** Analysis of the effect of H1C on IFN-β stimulated by IRF3 using real-time PCR. **(F)** Analysis of the effect of H1C or its mutants on IFN-β stimulated by IRF7 using real-time PCR. H1C silencing or expression and RIG-I, MAVS, TBK1, IKK-ξ, IRF3, and IRF7 expression levels were detected by western blotting (*p < 0.05, **p < 0.01, ***p < 0.001, the data were generated from three independent experiments).

Influenza virus can be recognized by RIG-I during infection and activates type-I interferon production by signaling transduction from RIG-I, MAVS, and TBK1/IKK-ξ complex to IRF3. To investigate how H1C regulates IFN-β, the IFN-β induction signaling pathway was analyzed. When co-transfected with the above factors and siRNA, H1C or its mutants on A549 cells, we found that IFN-β levels stimulated by all these factors were significantly influenced by H1C. For example, when stimulated with RIG-I, IFN-β production was significantly reduced by silencing H1C, but it was significantly increased by overexpression of H1C. In addition, K34A and K187A mutations significantly promoted the effect of H1C, while the T146A mutation significantly inhibited the effect (Figure 7A). Similar results were also observed when tested with MAVS (Figure 7B), TBK-1 (Figure 7C), IKK-ξ (Figure 7D), and IRF3 (Figure 7E). These findings indicated that H1C could affect IFN-β *via* IRF3. However, because IRF7 is also involved in IFN-β activation, the effect of H1C on IFN-β stimulated by IRF7 should be detected to confirm whether H1C regulates IFN-β specifically *via* IRF3. Unfortunately, IFN-β activated by IRF7 was slightly affected by overexpression or silencing of H1C (Figure 7F), indicating that H1C regulated IFN-β specifically *via* IRF3. The expression of each factor and the silencing efficiency or expression levels of H1C were detected by western blotting and are shown below.

### H1C Affects IRF3 Binding onto the IFN-β Promoter

**Figure 8:**
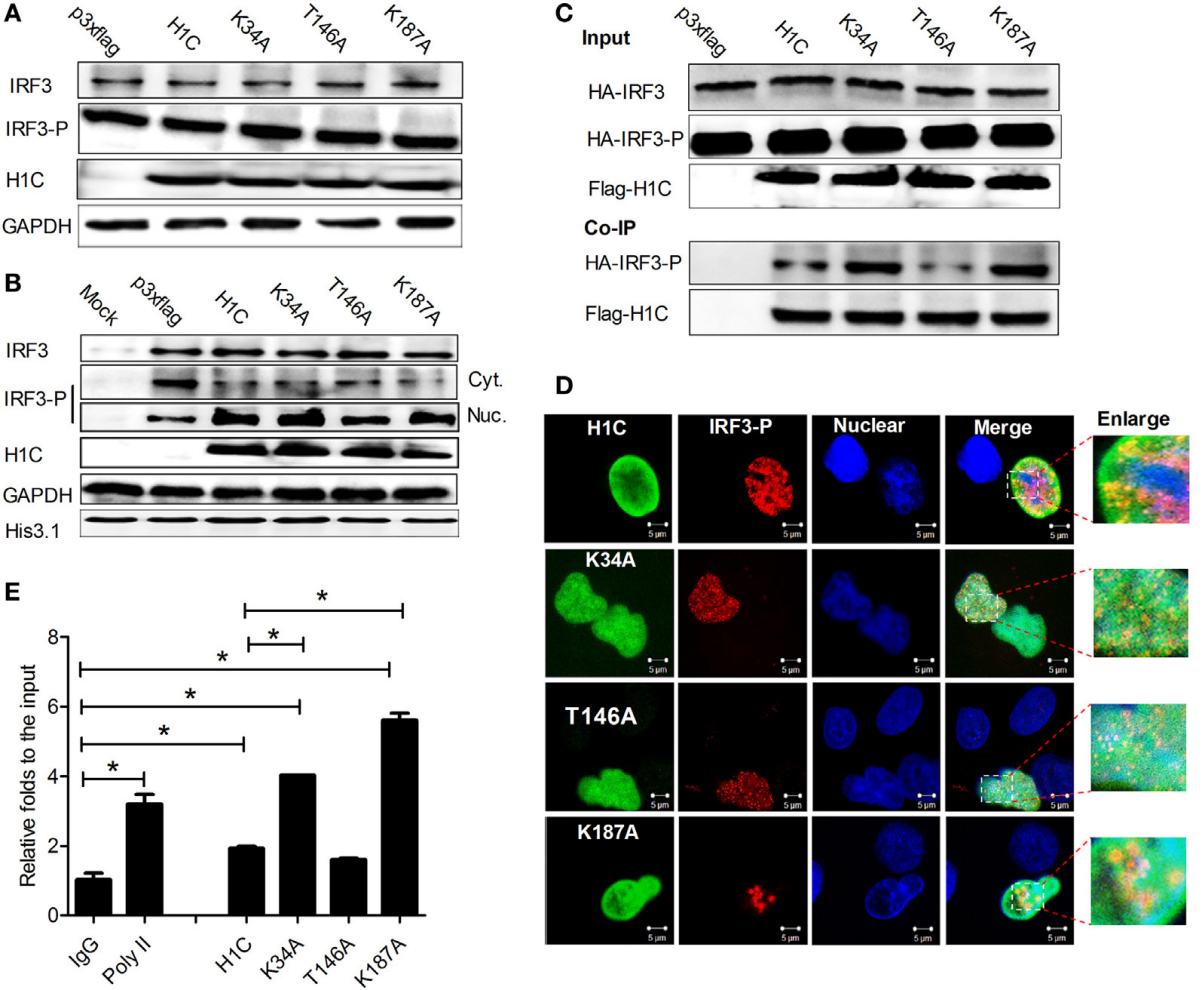
**The mechanism of H1C regulating interferon-β (IFN-β)**. **(A)** Analysis of the effects of H1C and its mutants on IRF3 and IRF3 phosphorylation. Human embryonic kidney 293 T (HEK293T) cells were overexpressed with H1C or its mutants and infected with Sendai virus (Sev) for an additional 12 h. IRF3 and IRF3 phosphorylation levels were then detected by western blotting using anti-IRF3 (sc-33642, Santa Cruz Biotechnology, Santa Cruz, CA, USA) or anti-IRF3-P antibody (Cat.#07-582-I, Millipore, Billerica, MA, USA). **(B)** Analysis of the effect of H1C on IRF3 nuclear transportation. HEK293T cells were treated as described above, and the cytoplasm and nuclear fractions were separated. IRF3 phosphorylation levels were then detected. **(C)** Detection of H1C–IRF3-P interactions; HEK293T cells co-transfected with HA-IRF3 and Flag-H1C or its mutants were infected with Sev for 12 h, and the co-immunoprecipitation (Co-IP) experiments were performed using anti-flag antibody and analyzed by western blotting using the anti-IRF3-P antibody to detect its interaction. **(D)** Investigation of co-localizations between H1C and IRF3-P. A549 cells cultured on slides were co-transfected with HA-IRF3 and Flag-H1C or its mutants and stimulated with poly (I:C) for 6 h, and immunofluorescence confocal microscopy was performed as previously described using anti-Flag mouse monoclonal antibody and anti-HA rabbit polyclonal antibody followed by immunostaining with FITC-labeled goat anti-mouse secondary antibody and Cy3-labeled goat anti-rabbit antibody. **(E)** Chromatin immunoprecipitation and quantitation PCR was performed to detect the effects of H1C on the capacity of IRF3 binding to the IFN-β promoter. HEK293T cells were co-transfected with HA-IRF3, and Flag-H1C or H1C mutants and chromatin immunoprecipitation assay were performed using am anti-HA antibody; the negative control was performed using the mouse IgG antibody, and the positive control was conducted using the anti-poly II antibody (*p < 0.05, the data were generated from one of three independent experiments).

During influenza virus infection, IRF3 is phosphorylated and transported into the nucleus to bind onto the IFN-β promoter and activate IFN-β production. Thus, to determine how H1C regulates IFN-β *via* IRF3, the effect of H1C on IRF3 phosphorylation was first detected. Unexpectedly, H1C showed a slight effect on IRF3 or IRF3 phosphorylation levels (Figure 8A). Thus, the effect of H1C on the nucleus transportation of phosphorylated IRF3 was investigated. These data showed that the level of phosphorylated IRF3 in the nucleus was significantly increased when overexpressing H1C, which was weakened by T146A. Unexpectedly, K34A and K187A mutation did not enhance this process. At the same time, we observed contrasting results with regard to the amount of IRF3-P in the cytoplasm, which was the opposite to findings observed in the nucleus and was much lower when overexpressing H1C or its mutants (Figure 8B). K34A and K187A mutants did not promote IRF3-P nuclear transportation compared with H1C, which indicated that H1C might regulate IFN-β by affecting IRF3 phosphorylation and nuclear transportation. To further explain this question, we determined whether H1C interacted with IRF3-P and affected its subsequent functions. To achieve this suppose, HA-IRF3 and Flag-H1C or its mutants were co-expressed and Co-IP experiments were performed in HEK293T cells. To our surprise, the data showed that H1C interacted with IRF3-P. Interestingly, the methylation mutants (K34A, K187A) showed a stronger ability to interact with IRF3-P than H1C wild type, and the phosphorylation mutation appeared weakened this interaction (Figure 8C). Further experiments showed that H1C and its mutants co-localized with IRF3-P in the nucleus (Figure 8D). This finding suggested that H1C might affect IRF3 binding onto the IFN-β promoter *via* its interaction. To confirm this speculation, HA-IRF3 and Flag-H1C or its mutants were co-expressed and chromatin immunoprecipitation was performed in HEK293T cells. The data showed that K34A and K187A mutants significantly enhanced the ability of IRF3 to bind onto IFN-β promoter (Figure 8E). Taken together, these data revealed that H1C regulated IFN-β by affecting IRF3 nuclear transportation and IFN-β promoter binding.

### NS2 Inhibits IFN-β Enhanced by H1C

**Figure 9:**
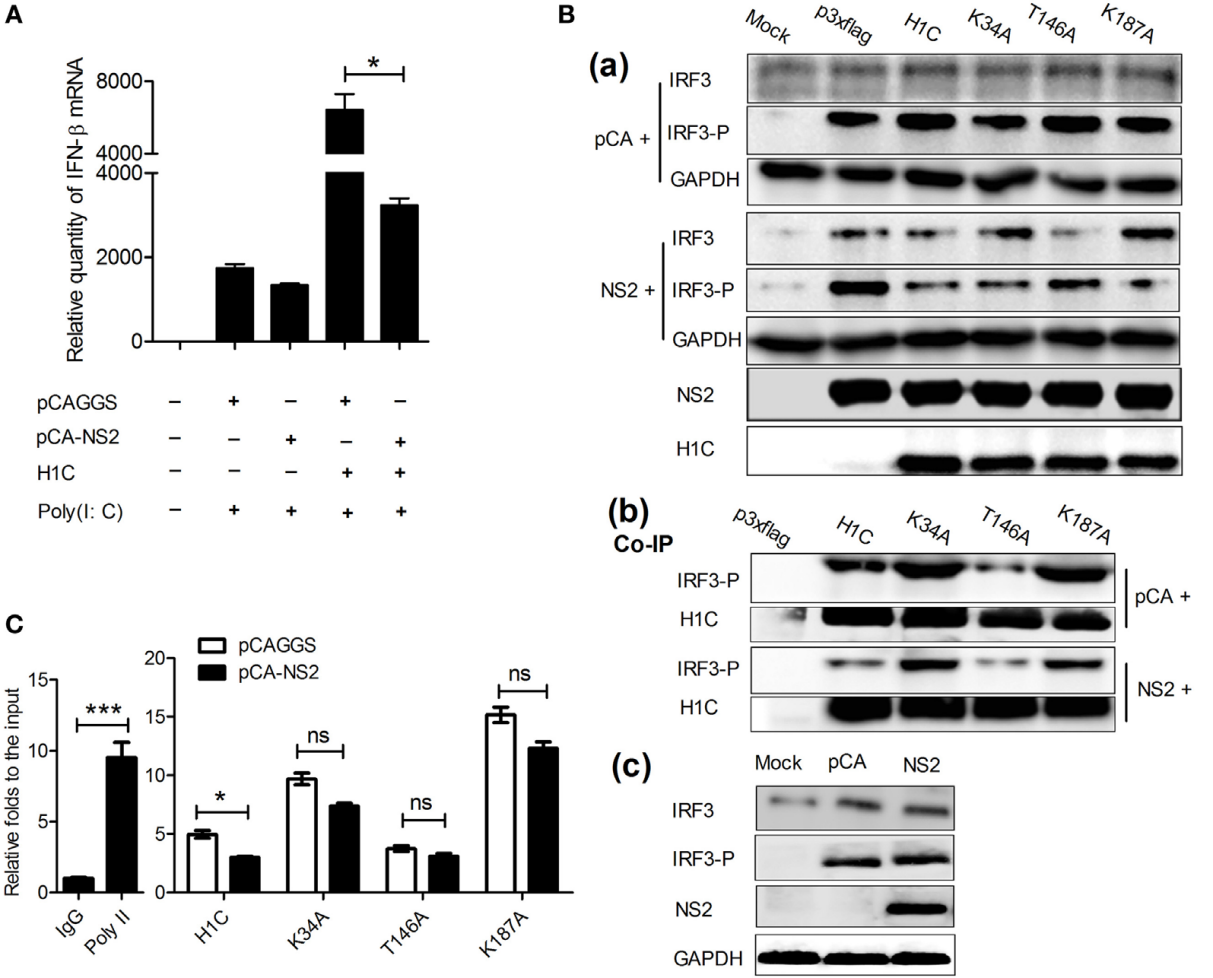
**NS2 inhibits interferon-β (IFN-β) induced by H1C**. **(A)** Investigation of the effect of NS2 on IFN-β stimulated by H1C. A549 cells were transfected with pCAGGS and pCAGGA-NS2 individually, or co-transfected with p3xflag or H1C, then stimulated by poly (I:C) (20 nmol/mL) for 6 h, and IFN-β mRNA was detected by real-time PCR. **(B)** NS2 affects IRF3 phosphorylation and H1C–IRF3-P interaction. (a) Human embryonic kidney 293 T (HEK293T) cells co-expressed with NS2 and H1C or its mutants were infected with Sendai virus (Sev) for 10 h. Next, the cells were lysed, and IRF3 and IRF3-P levels were detected by western blotting, respectively; (b) cells were treated as described above, and co-immunoprecipitation (Co-IP) experiments were performed to detect the effect of NS2 on the H1C–IRF3-p interaction; (c) detection of the effect of NS2 on IRF3 and IRF3 phosphorylation. Human embryonic kidney 293 T cells were transfected with pCAGGS or pCAGGS-NS2 and infected with Sev for 12 h. IRF3 and IRF3 phosphorylation levels were detected by western blotting. **(C)** Analysis of the effects of NS2 on the ability of IRF3 to bind to the IFN-β promoter by chromatin immunoprecipitation and quantitation PCR. HEK293T cells co-transfected with IRF3, NS2, and H1C or its mutants and stimulated by Sev, and the chromatin immunoprecipitation assay was performed using anti-IRF3-P antibody. The negative control was performed using the mouse IgG antibody, and the positive control was performed using the anti-poly II antibody (*p < 0.05, ***p < 0.001, the data were generated from one of three independent experiments).

Would NS2 be involved in the H1C regulation of IFN-β? To determine this, NS2 was expressed individually or co-expressed with H1C and the effect of NS2 on IFN-β was detected. The data showed that NS2 slightly affected IFN-β production when overexpressed alone, but it significantly reduced IFN-β when co-expressed with H1C (Figure 9A), indicating that NS2 is involved in the regulation of H1C on IFN-β. Thus, the effect of NS2 on IRF3 phosphorylation when co-expressed with H1C was evaluated. The result showed that NS2 significantly decreased IRF3 phosphorylation levels when co-expressed with H1C or its mutants compared with the vector. But, when NS2 was expressed with H1C, the IRF3 phosphorylation levels was sharply decreased, and similar results were found in the K34A and K187A mutant groups. However, the IRF3 levels were higher compared to the vector groups (Figure 9B, a). In addition, NS2 slightly affected IRF3 expression or phosphorylation when overexpressed alone (Figure 9B, c). Because H1C interacts with both NS2 and IRF3-P, which suggests that NS2 might affect H1C–IRF3 interaction; thus, NS2 was co-expressed with H1C and IRF3, and Co-IP experiments were performed to detect the potential effect of NS2 on H1C–IRF3 interaction. These results showed that NS2 reduced the interactions between IRF3 and H1C or its mutants (Figure 9B, b) indicating that NS2 might affect IRF3 binding onto the IFN-β promoter when expressed with H1C. Further experiments were performed to investigate this question, and these result showed that NS2 significantly inhibited the ability of IRF3 to bind onto the IFN-β promoter when co-expressed with H1C, and it also inhibited the ability of IRF3 to bind onto the IFN-β promoter when co-expressed with K34A, T146A or K187A but without significance (Figure 9C), indicating that inhibition of NS2 on IFN-β induced by H1C relied on the methylation and phosphorylation of H1C.

## Discussion

The innate immune response is an important defense for the host against viral infection during the early stage, which includes the activation of interferon, inflammation, chemokines, and other antiviral factors. Here, we find that H1C not only affects IFN-β but also TNF-α and CXCL10, indicating that H1C is a multifunctional factor. TNF-α plays crucial roles in the regulation of inflammation, and it induces apoptosis by activating the JNK/Caspase signaling pathway. Influenza virus replication can lead to cell apoptosis and inflammation; and apoptosis is not conduce to influenza virus replication at the early stage while it promotes virus release from the cell at the later stage; in addition, inflammation can accelerates host cell to clear the infection (27). A recent study showed that IKK inhibits TNFα-induced apoptosis *via* two distinct but cooperative mechanisms: activation of the survival factor NF-κB and inactivation of the proapoptotic BH3-only BAD protein (28). These findings suggest that H1C is involved in the regulation of apoptotic and inflammation by regulating TNF-α or other pathways related to TNF-α, thereby affecting influenza viral replication. In addition, CXCL10 exhibits chemo-attractive effects on macrophages, T cells, NK cells, and dendritic cells, which promotes the host to clear infection. And CXCL10 binds to a common receptor chemokine (C–X–C motif) receptor 3 (CXCR3) and activates multiple functions of CD8^+^ T cells (29). Moreover, CXCR3 has been found to be important in the pathogenesis of several viral infections, including influenza (30). These findings suggest that H1C can regulates influenza viral replication *via* different methods, and it also suggests that H1C may be involved in the regulation of other viruses.

**Figure 10:**
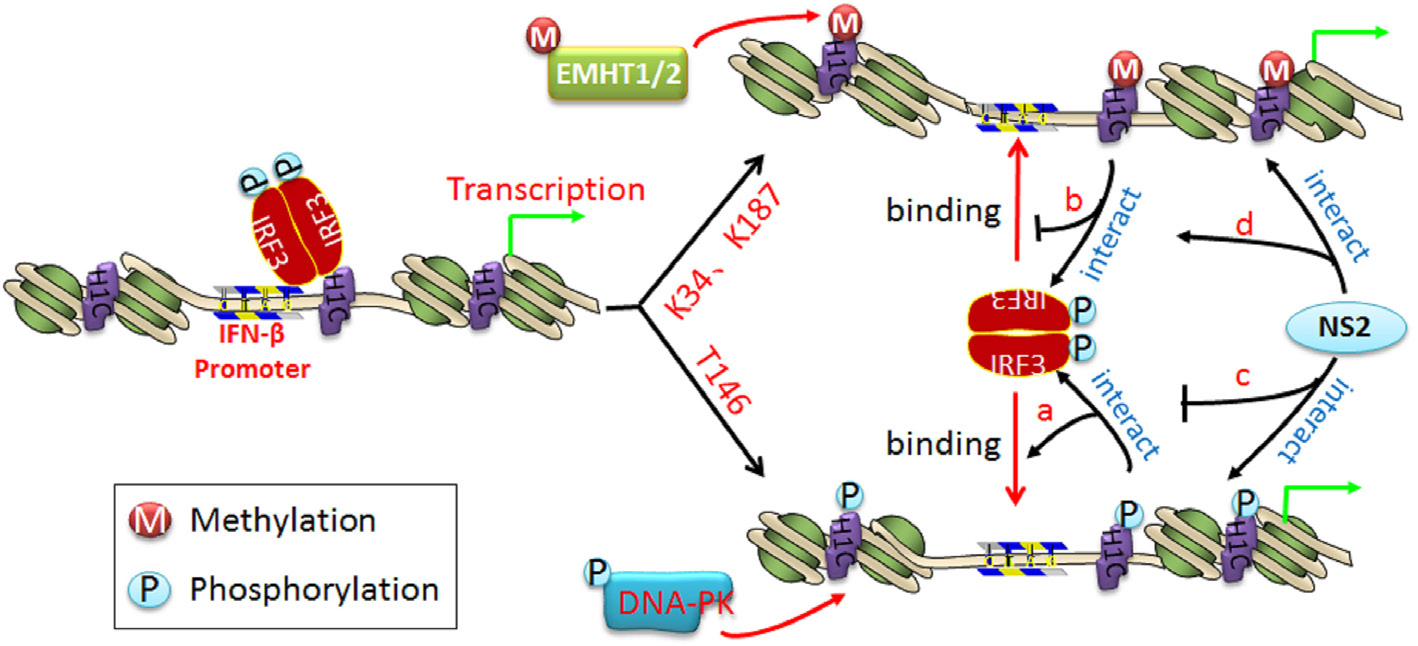
**The schematic diagram of H1C regulating interferon-β (IFN-β) and the function of NS2 in it**. When IRF3 is phosphorylated and transported into the nucleus, it recruits co-factors and binds to the IFN-β promoter to start the IFN-β transcription. (a) When H1C is phosphorylated, it opens the nucleosome and makes it looser, which promotes IRF3 binding to the IFN-β promoter and induces more IFN-β production; (b) when H1C is methylated, it brings the nucleosome structure closer, which inhibits IRF3 binding to the IFN-β promoter and decreases IFN-β production; (c) in addition, NS2 interacts with phosphorylated H1C, which reduces the H1C–IRF3 interaction and results in an inhibition of IRF3 binding to the IFN-β promoter and less IFN-β production; (d) NS2 interacts with methylated H1C, which promotes the interaction between H1C and IRF3 and enhances the inhibition of IFN-β production.

Three distinct families of transcription factors, including nuclear factor-κB (NF-κB), activating transcription factor 2/c-Jun, and IRF3, are involved in IFN-β transcription (31). Of these, IRF3 plays a crucial role in the induction of IFN-β. H1C slightly affects IRF3 phosphorylation but promotes IRF3 nuclear transportation, which increases IFN-β production. However, K34A and K187A mutants appear to have little effect on IRF3 nuclear transportation compared with H1C wild type but increase the ability of IRF3 binding onto the IFN-β promoter, indicating that H1C regulates IFN-β by affecting IRF3 nuclear transportation and IRF3 binding onto the IFN-β promoter. H1C might recruit IRF3 binding onto IFN-β promoter by interacting with IRF3. It may change the structure of the nucleosome *via* its simultaneous phosphorylation and methylation; thus, H1C phosphorylation may release the nucleosome and promote polymerase II (poly II) (32) and IRF3 binding onto the IFN-β promoter to start transcription (Figure 10, a). Conversely, H1C methylation may make the nucleosome tighter, preventing IRF3 binding onto the IFN-β promoter (Figure 10, b) and suggesting that H1C is involved in epigenetic regulation of the gene *via* its phosphorylation and methylation. Thus, the equilibrium of H1C phosphorylation and methylation is important for its effect on IFN-β. Similar studies have also found that histones are involved in epigenetic regulation; for example, HP1-beta genome-wide localization follows H3K9me3-enrichment, and the artificial bridging of chromatin fibers is sufficient to maintain cellular heterochromatic conformation (33, 34). DNA methylation plays an unexpected dual role at enhancer regions and is anti-correlated focally at transcription factor-binding sites but positively correlated globally with the active H3K27ac mark to ensure structural enhancer integrity (35, 36).

In addition, it has been found that mice embryos lacking the three H1 subtypes (H1C, H1D, and H1E) die by mid-gestation with a broad range of defects (37), and mutations in HIST1H1 B-E closely related to the pathogenesis of follicular lymphoma (38). These findings suggest that the mutations and modifications of H1C involve in a wide range of regulations. Moreover, a recent study has identified a H1C SNP variant A18V in MCF-10A cells (39) while Linder found that alanine at position 17 in H1.2 was replaced by valine in K562 erythroleukemic cells (40), but the function of these variants in innate immunity were not clear.

On the other hand, a recent study found that the NS1 protein of influenza A H3N2 subtype exhibits a histone-like sequence (histone mimic), which is used by the virus to target the human PAF1 transcription elongation complex (hPAF1C), resulting in the suppression of hPAF1C-mediated transcriptional elongation and contributing to the suppression of the antiviral response (41). However, it is a unique mechanism and not suit for the other influenza virus strains. In this study, NS2 reduces IRF3 phosphorylation and H1C–IRF3 interaction when co-expressed with H1C, indicating that NS2 is involved in the regulation of IFN-β by decreasing IRF3 phosphorylation and comparatively interacting with H1C or its mutants, thereby reducing the H1C–IRF3 interaction and further affecting IRF3 binding onto the IFN-β promoter as a result of decreases in IFN-β production (Figure 10, c and d). However, the ability of IRF3 to bind to the IFN-β promoter was only slightly decreased by NS2 when K34, T146, and K187 were mutated. This may because K34A and K187A mutants have stronger ability to promote IRF3-P to bind onto IFN-β promoter thereby produce much higher IFN-β; thus, when NS2 co-expressed with K34A or K187A mutants, it could only decrease the ability of IRF3 binding onto IFN-β promoter to some degree though it can decrease IRF3-P levels and H1C–IRF3 interactions significantly. And when co-expressed with T146A mutant, NS2 showed slightly inhibition to the ability of IRF3 to bind onto IFN-β promoter because the T146A mutation could significantly decrease the ability of IRF3 to bind onto IFN-β promoter itself. These findings indicate that NS2 inhibits IFN-β induced by H1C and is dependent on the phosphorylation and methylation of H1C to some degree.

In summary, we identified a novel function of H1C in the innate immune response and uncovered the mechanism. We also demonstrated that NS2 is involved in this process, revealing a new approach for influenza A virus to evade the innate immune response.

## Author Contributions

MJ and PQ designed the study; XKL and CY performed the experiments; XKL wrote the manuscript; and YH, EL, XL, LZ, ZZ, AZ, HZ, and HC analyzed the data.

## Conflict of Interest Statement

The authors declare that the research was conducted in the absence of any commercial or financial relationships that could be construed as a potential conflict of interest.
